# Development and deployment of a nationwide predictive model for chronic kidney disease progression in diabetic patients

**DOI:** 10.3389/fneph.2023.1237804

**Published:** 2024-01-08

**Authors:** Zhiyan Fu, Zhiyu Wang, Karen Clemente, Mohit Jaisinghani, Ken Mei Ting Poon, Anthony Wee Teo Yeo, Gia Lee Ang, Adrian Liew, Chee Kong Lim, Marjorie Wai Yin Foo, Wai Leng Chow, Wee An Ta

**Affiliations:** ^1^ Integrated Health Information Systems (IHIS), Singapore, Singapore; ^2^ Mount Elizabeth Novena Hospital, Singapore, Singapore; ^3^ National Health Group Polyclinics, Singapore, Singapore; ^4^ Department of Renal Medicine, Singapore General Hospital, Singapore, Singapore; ^5^ Epidemiology and Disease Control Division, Ministry of Health, Singapore, Singapore

**Keywords:** chronic kidney disease, CKD progression, diabetes, kidney function deterioration, machine learning, prediction

## Abstract

**Aim:**

Chronic kidney disease (CKD) is a major complication of diabetes and a significant disease burden on the healthcare system. The aim of this work was to apply a predictive model to identify high-risk patients in the early stages of CKD as a means to provide early intervention to avert or delay kidney function deterioration.

**Materials and methods:**

Using the data from the National Diabetes Database in Singapore, we applied a machine-learning algorithm to develop a predictive model for CKD progression in diabetic patients and to deploy the model nationwide.

**Results:**

Our model was rigorously validated. It outperformed existing models and clinician predictions. The area under the receiver operating characteristic curve (AUC) of our model is 0.88, with the 95% confidence interval being 0.87 to 0.89. In recognition of its higher and consistent accuracy and clinical usefulness, our CKD model became the first clinical model deployed nationwide in Singapore and has been incorporated into a national program to engage patients in long-term care plans in battling chronic diseases. The risk score generated by the model stratifies patients into three risk levels, which are embedded into the Diabetes Patient Dashboard for clinicians and care managers who can then allocate healthcare resources accordingly.

**Conclusion:**

This project provided a successful example of how an artificial intelligence (AI)-based model can be adopted to support clinical decision-making nationwide.

## 1 Introduction

Chronic kidney disease (CKD) is a major complication arising from diabetes, and around 25%–40% of patients with diabetes develop diabetic kidney disease (1). Diabetes is not only one of the most common diseases but also the leading cause of kidney failure. In Singapore, diabetes accounts for two-thirds of cases of end-stage kidney failure (ESKF) requiring dialysis, with the prevalence being one of the highest in the world according to the United States Renal Data System (USRDS)’s data (https://adr.usrds.org/2021/end-stage-renal-disease/11-international-comparisons). Diabetes leads to kidney damage via two main pathways: chronic hyperglycemia and the activation of the renin–angiotensin system. These processes ultimately induce glomerular sclerosis, albuminuria, and kidney impairment (2). CKD is projected to be the fifth leading cause of death globally by 2040 (3). Because it is often asymptomatic in the early stages, CKD is usually diagnosed late. The treatment for patients with ESKF requires substantial resources and is still a great challenge to a healthcare system that is under resource limits and budget control. The patients themselves also suffer from a significant loss of quality of life and from the financial burdens affecting them as a result of their condition.

It is critical to identify patients who are at high risk of kidney function deterioration to avert or delay their progression to ESKF. These patients could be treated in the early stages and be engaged early on in their long-term kidney care plans. The Nephrology Evaluation, Management and Optimization (NEMO) program was introduced to optimize renal protection treatment earlier within the primary care setting. Compared with diabetic patients not enrolled in NEMO, the patients in the NEMO program had a 28% lower rate of kidney function deterioration (https://www.healthhub.sg/a-z/medical-and-care-facilities/nemo-programme-for-diabetic-kidney-patients). However, the NEMO program focused only on patients from one polyclinic cluster in Singapore and addressed only one treatment parameter. Consequently, the Holistic Approach in Lowering and Tracking Chronic Kidney Disease (HALT-CKD) program, which is comprised of nephrologists from all public hospitals in Singapore, was launched in 2017 as an enhanced and extended version of the NEMO program. The HALT-CKD national program targets and tracks a much broader cohort, that is, all CKD patients from stage 1 to stage 4 in Singapore. It is also carried out at all polyclinics across Singapore and aims to identify and control evidence-based risk factors of CKD to delay CKD progression.

A prediction method of CKD progression has been studied in some western countries’ populations to assist in the early intervention of patients. Tangri et al. (4) developed a risk model of CKD progression to ESKF in the Canadian population and found a list of important indicators—age, sex, estimated glomerular filtration rate (eGFR), and levels of urine albumin, serum calcium, serum phosphate, serum bicarbonate, and serum albumin. Later, this model was evaluated and recalibrated for multinational cohorts (5). The method of Tangri et al. was also applied to predict the probability and the timing of kidney failure that requires kidney replacement therapy (6). Ramspeck et al. (7) selected 11 prediction models on kidney failure and validated them on two large cohorts in Europe. The model’s performance on the validation cohorts was considerably worse than on the original cohorts due to the narrower patient mix of the validation cohorts. This result indicates the necessity of retraining and even redesigning the CKD prediction model when applying it to populations with different ethnicities.

Low et al. (8) followed 1,582 patients with type 2 diabetes mellitus, from 2002 to 2014, and developed a logistic regression model for CKD progression specifically for diabetic patients in one Singapore hospital. In recent years, machine-learning algorithms, such as random forest and XGBoost, were also used to create the risk models of CKD progression, which showed a potential for improvement from statistical methods (9, 10).

However, there are some limitations in the previous studies. First, the study population did not cover the different ethnic groups in Singapore and was not representative of all diabetic patients in the country. Second, there was no clinical validation to confirm the viability of the models and to demonstrate their clinical usefulness in practice. Some studies did not even have validation datasets to check the stability of the model’s performance.

In the current study, we developed a predictive model for CKD progression in 5 years for diabetic patients in Singapore. Our study used the nationwide medical data of patients in Singapore by utilizing the two national systems: the National Diabetes Database (NDD) and the Business Research Analytics Insights Network (BRAIN). Our study result has been validated technically and clinically by nephrologists from public hospitals, hence the endorsement from the HALT-CKD program’s committee to incorporate it into its action plan.

## 2 Materials and methods

### 2.1 Data collection and model development/deployment platform

The patient data used in this study were from the National Diabetes Database (NDD) within the Business Research Analytics Insights Network (BRAIN) platform.

#### 2.1.1 National Diabetes Database

**Figure 1:**
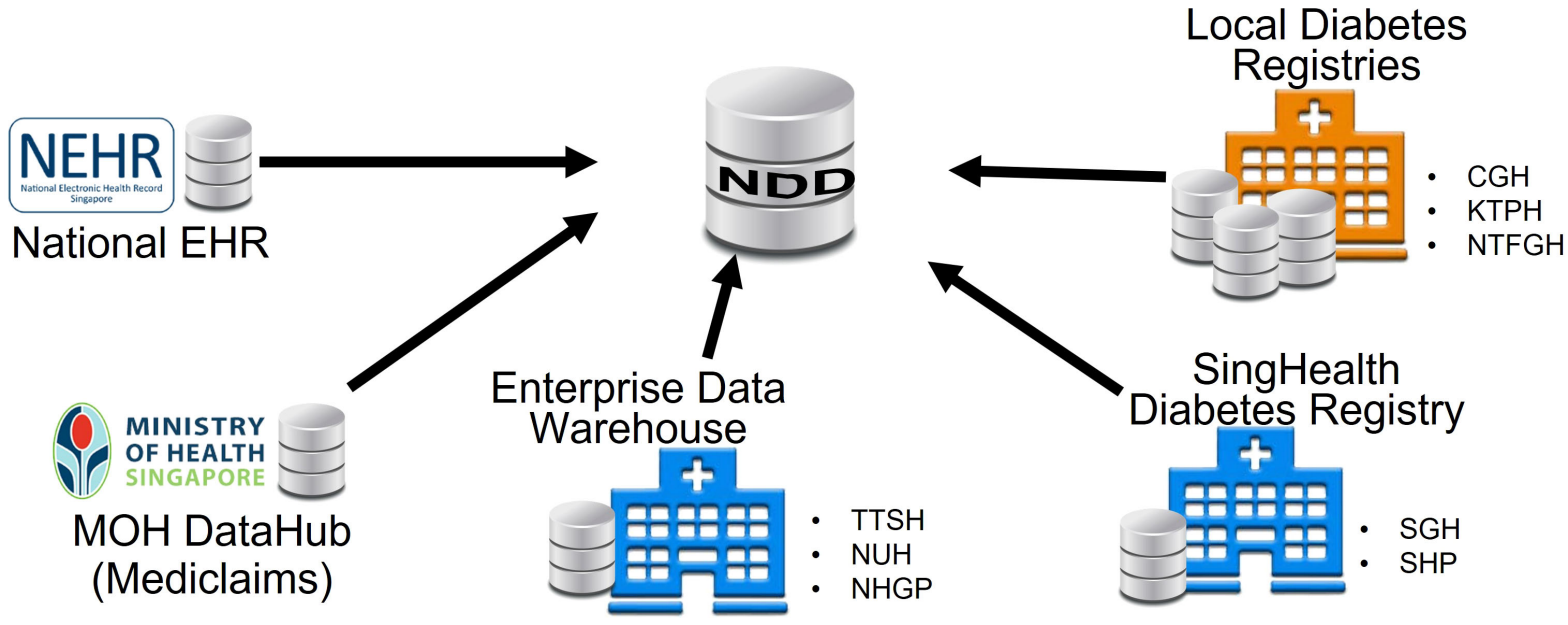
Formation of the National Diabetes Database.

The NDD consolidated data from multiple repositories across Singapore. It was jointly developed by the Ministry of Health (MOH) and the Integrated Health Information Systems (IHIS) in Singapore to support the national “War on Diabetes” initiative, which aims to enhance citizens’ awareness of diabetes and to create a supportive environment to prevent and manage diabetes well (https://www.moh.gov.sg/wodcj). Prior to the creation of the NDD, each hospital had its own registry of diabetic patients. The diabetic patients and their data were sat in silos, as shown in **Figure 1**. The data may be overlapped and inconsistent due to different data sources and criteria. The NDD standardized the criteria and integrated data from multiple sources and kept it updated with the new information. It aims to facilitate outreach, policy intervention, and better clinical management for diabetic patients.

The NDD has provided high-quality data on diabetic patients in Singapore since 2011. It covers nine major public hospitals in Singapore: Changi General Hospital (CGH), Singapore General Hospital (SGH), KK Women’s and Children’s Hospital (KKH), Seng Kang Hospital (SKH), Khoo Teck Puat Hospital (KTPH), Tan Tock Seng Hospital (TTSH), National University Hospital (NUH), Ng Teng Fong General Hospital (NTFGH), and Alexandra Hospital (AH). It also covers three polyclinic clusters in Singapore: Singhealth Group Polyclinics (SHP), National Healthcare Group Polyclinics (NHGP), and National University Polyclinics (NUP). The data include demographics, events, laboratory tests, diagnoses, medications, foot screenings, eye screenings, procedures, clinical measurements, finance, referrals, and mortality (see details in **Supplementary Figure 1**).

#### 2.1.2 Business Research Analytics Insights Network

The NDD and the CKD prediction tools reside in the BRAIN (11). The CKD prediction model was developed and deployed in the BRAIN platform. The relevant data and predicted scores are stored and aggregated in the NDD. These data are used to produce dashboards for policymakers and clinicians (see examples in **Supplementary Figures 2**, **3**).

### 2.2 Study base cohort for the model’s development

**Figure 2:**
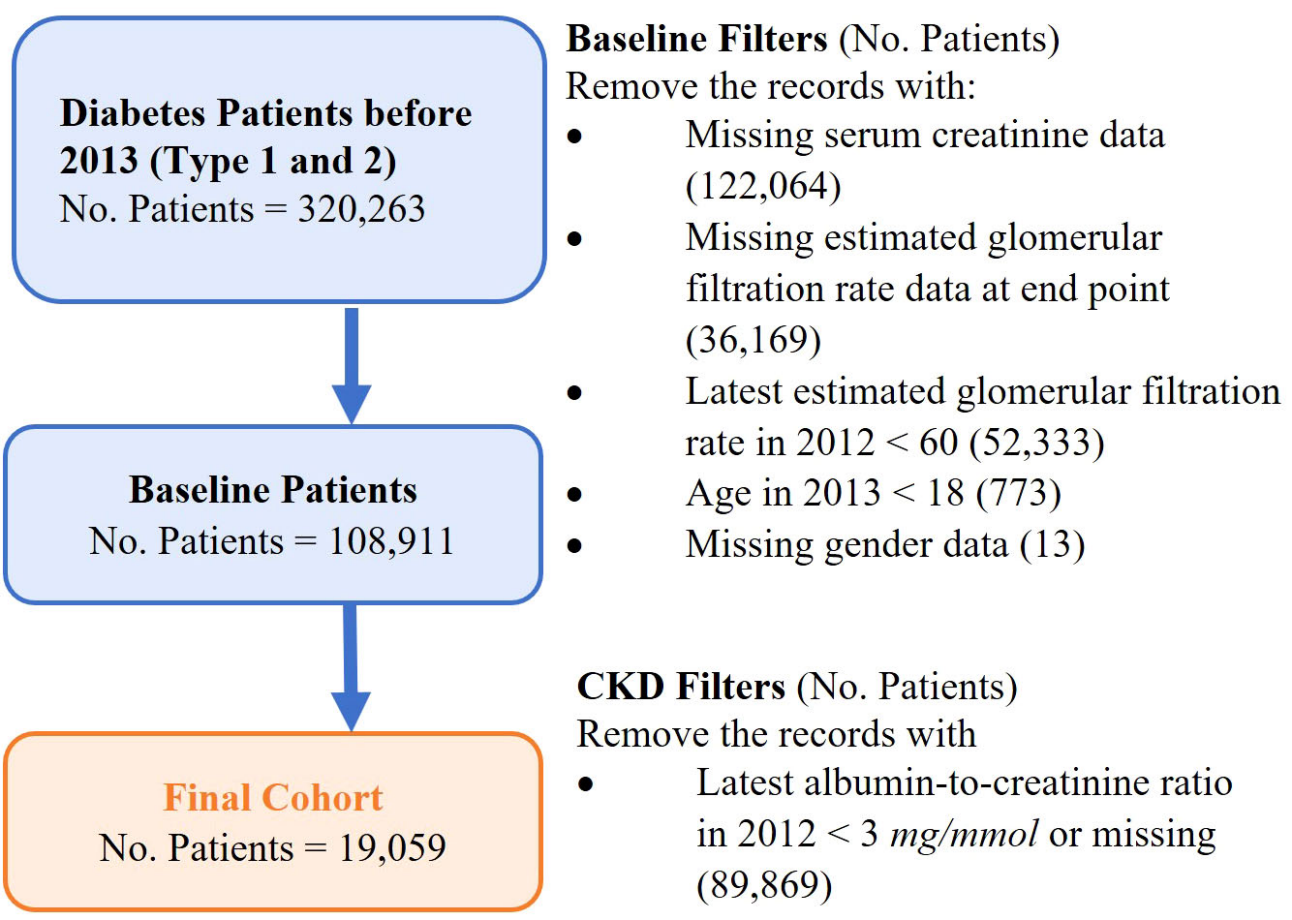
Definition of the study base cohort for the model’s development.

A retrospective study cohort consists of adult patients diagnosed with diabetes and in the early stages of CKD before 1 January 2013 in Singapore. The study cohort was used to develop a predictive model on CKD progression. **Figure 2** shows the formation of the study cohort with the exclusion criteria: (i) patients not having type 1 diabetes or type 2 diabetes before 1 January 2013, (ii) patients not in CKD stage 1 or stage 2 in 2012, (iii) patients aged < 18 years in 2013, and (iv) patients with missing gender data. The definitions of the different stages of CKD were based on the Kidney Disease Improving Global Outcome Guidelines (KDIGO) with slight modifications (refer to **Supplementary Material** for details). Patients who were at CKD stages 3–5 were identified by two estimated glomerular filtration rate (eGFR) readings that were more than 90 days apart to exclude acute kidney injury episodes. The historical data of medication and laboratory tests were extracted to analyze patients’ medical and clinical conditions^1^.

### 2.3 Predictive model variables and feature engineering

The dependent variable of the predictive model was defined as a patient’s 5-year risk of CKD progression. That is, whether a patient’s kidney damage will deteriorate into CKD stage 3 or above in 5 years. Specifically, the model was trained on the study base cohort to predict on 1 January 2013 their risk of CKD progression in the next 5 years until 1 January 2018.

Independent variables of the model encompass five categories: sociodemographic characteristics, blood pressure, diagnosis, laboratory tests, and medication. Feature engineering was conducted to convert the study data into these variables based on previous studies of CKD progression and input from clinicians and care managers. There were 177 variables generated initially, and feature selection was performed using the backward feature selection method to choose the most relevant features while maximizing the model’s performance (12, 13) during model training. The feature selection process was done by repeatedly removing the variables that were not important according to the feature importance of the XGBoost model or not contributing to the model’s performance, until the variables in the final models were all important in prediction, and the removal of any variable would lead to a significant drop in the model’s performance. There were 25 variables selected to be included in the model.

### 2.4 Machine-learning model development

We used the Extreme Gradient Boosting algorithm (XGBoost) to build the predictive model and grid search to fine-tune hyper-parameters. The hyper-parameters included the maximum depth of each decision tree (i.e., *max_depth*), the minimum sum of instance weight (hessian) needed in a child (i.e., *min_child_weight*), and the number of decision trees in the final model (i.e., *nrounds*). Our model using XGBoost could handle the missing data directly due to the sparse-aware split finding (14). The model was developed in R (R is a programming language for statistical computing, created by Ross Ihaka and Robert Gentleman).

The study cohort was randomly split into two datasets by patient ID: a training dataset for the model’s development and a test dataset for the model’s validation. **Table 1** describes patients’ distribution in the two datasets. To avoid the overfitting problem and to fine-tune the hyper-parameters, threefold cross-validation was applied to the training dataset. Cross-validation separated the data into several partitions, trained on different combinations of partitions, and evaluated the remaining partitions. The model’s performance was measured by three metrics: area under the curve (AUC), positive predictive value (PPV), and sensitivity when PPV and sensitivity have a trade-off relationship influenced by the risk score threshold.

**Table 1:** Distribution of patients and CKD progression in the different datasets.

Data	Number of patients		CKD progression into stage 3 or above in 5 years	
		%	Number of patients	%
Training	14,240	75%	5,300	37.2%
Test	4,810	25%	1,801	37.4%
Total	19,050	100%	7,101	37.3%

### 2.5 Technical and clinical validation of the developed model

Technical validation of the model developed was performed using a new cohort with 6-month shift test data. The new cohort consisted of patients filtered using the same exclusion criteria as the base cohort but shifting the ending date to 30 June 2013, instead of 31 December 2012. The patients in the base cohort were removed so that the new cohort had no overlap with the base cohort. The technical cohort contained 5,490 patients and their risk of CKD progression in the next 5 years until 30 June 2018 was predicted using our developed model.

One-year shift data (2013 cohort) were generated for clinical validation by shifting the ending date of the cohort to 31 December 2013 and removing the patients overlapped with the base cohort or the technical validation cohort. One thousand patients were randomly selected from the 2013 cohort, and the same features used by our model were provided to 20 clinicians across three polyclinics groups. The clinicians were asked to assess the patients’ risk of CKD progression in the next 5 years; we then compared the clinicians’ assessment with our model’s prediction.

The model package and the development script are available at: https://github.com/beverly0005/CKD/tree/main.

## 3 Results

In the current study, we developed a model for CKD progression in diabetic patients. Our study cohort contained 19,050 adult patients diagnosed with diabetes and CKD stages 1 and 2 before 2013; 37.3% of them deteriorated into CKD stage 3 or above in 5 years. **Table 2** shows the descriptive summary of the study cohort. The training, test, and validation datasets all showed similar characteristics as shown in **Table 2**.

**Table 2:** Descriptive statistics of the study cohort.

Variable Study cohort, *N* = 19,059	% Missing	% Cohort	
Male (yes, 1; no, 0)	0	48.7%	
Ethnicity: Chinese (yes, 1; no, 0)	0	69.3%	
Ethnicity: Indian (yes, 1; no, 0)	0	12.5%	
Ethnicity: Malay (yes, 1; no, 0)	0	14.4%	
	% missing	Mean (SD)	Interquantile, Q1, Q3
Age in 2013 (years)	0	61.6 (11.4)	54.0, 69.0
Diabetes duration (years)	0	7.5 (5.9)	3.0, 10.0
Latest hemoglobin A_1C_ result before 2013 (%)	0.2	7.9 (1.6)	6.8, 8.5
Prior hemoglobin A_1C_ result before 2013 (%)	5.9	7.9 (1.6)	6.8, 8.5
Latest albumin urine result before 2013 (mg/g)	2.1	213.3 (479.7)	36.4, 176.6
Latest glucose result before 2013 (mmol/L)	3.1	8.5 (3.4)	6.4, 9.6
Latest hemoglobin result before 2013 (g/dL)	48.3	13.0 (1.7)	11.9, 14.2
Prior hemoglobin result before 2013 (g/dL)	77.3	12.6 (1.8)	11.4, 13.9
Latest urine albumin-to-creatinine ratio before 2013 (mg/mmol)	0	29.8 (83.6)	4.7, 21.8
Latest albumin serum result before 2013 (g/L)	67.8	38.3 (6.0)	35.0, 42.0
Prior albumin serum result before (g/L)	87.8	37.6 (5.9)	34.0, 42.0
Latest eGFR before 2013 (mL/min/1.73m^2^)	0	88.2 (16.1)	75.0, 100.0
Prior eGFR before 2013 (mL/min/1.73m^2^)	50.8	98.7 (41.3)	76.0, 103.0
Latest alanine aminotransferase result before 2013 (U/L)	3.8	27.2 (17.6)	16.0, 33.0
Latest triglyceride result before 2013 (mmol/L)	2.3	1.6 (1.1)	1.1, 1.9
Latest high-density lipoprotein result before 2013 (mmol/L)	1.6	1.3 (0.4)	1.0, 1.5
Latest low-density lipoprotein result before 2013 (mmol/L)	2.1	2.5 (0.8)	2.0, 2.9
Latest total cholesterol result before 2013 (mmol/L)	2.3	4.5 (1.0)	3.9, 5.0
Number of distinct medicines (ATC codes) for the alimentary tract or metabolism dispensed in 2012	0	3.6 (2.3)	2.0, 5.0
Number of distinct medicines (ATC codes) for cardiovascular system dispensed in 2012	0	3.1 (1.6)	2.0, 4.0

eGFR, estimated glomerular filtration rate.

Following our feature selection process, the original 177 variables were narrowed down to four categories of variables and used for training our CKD model, as elaborated below. The sociodemographic characteristics included age, gender, and race. The diagnosis variable included diabetes duration, which was the number of days from the earliest diabetes diagnosis date to the date of prediction, i.e., 1 January 2013. The variables derived from the laboratory test history included measurements of hemoglobin A_1C_, albumin urine, glucose, hemoglobin, the urine albumin-to-creatinine ratio, albumin serum, eGFR, alanine aminotransferase, triglycerides, high-density lipoprotein, low-density lipoprotein, and total cholesterol. The glomerular filtration rate (GFR) is an important metric that reflects kidney function, but measurement requires a complicated and lengthy procedure. The eGFR is an approximate to glomerular filtration rate. It is calculated using the CKD-EPI equation based on serum creatinine, age, gender, and ethnicity (non-Black www.kidney.org/content/ckd-epi-creatinine-euqation-2009). The statistics of these laboratory tests are shown in **Table 2**. The “prior” laboratory readings in **Table 2** refer to the test result closest to but at least 90 days before the latest laboratory reading before 1 January 2013. This gives an indication of the magnitude of the change in the laboratory results and provides a sense of the progression of the patients’ clinical conditions and parameters.

The medication data were standardized into the groups from the Anatomical Therapeutic Chemical Classification (ATC) of which the first level contains 14 main anatomical and pharmacological groups (15). For example, an ATC code with the first level “A” tells that the drug is related to the alimentary tract or metabolism, while the ATC code with the first level “C” tells that the drug acts on the cardiovascular system.

**Figure 3:**
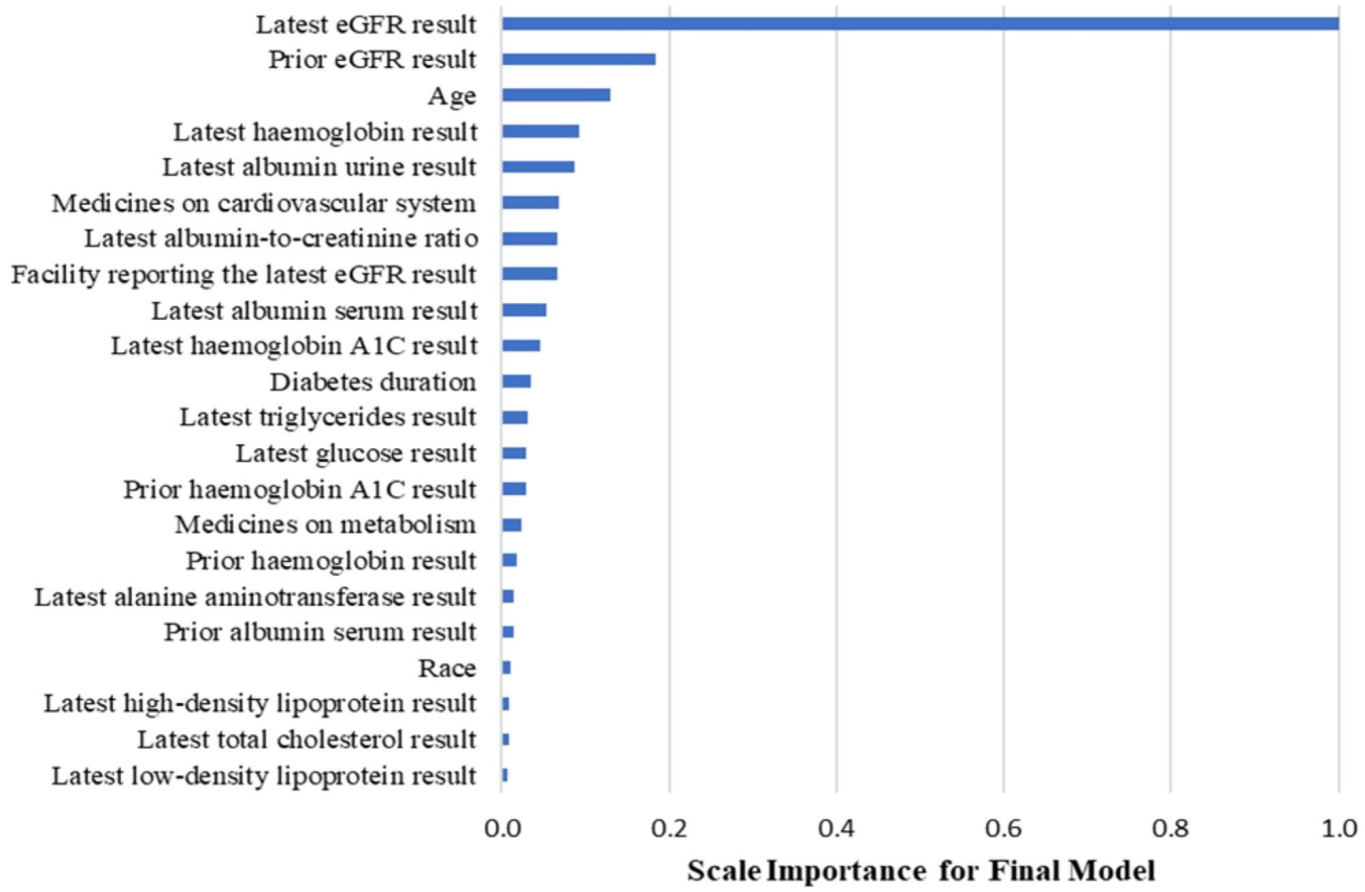
Variable importance in the CKD model.

**Figure 3** displays the important predictive factors for the CKD progression in our model. Consistent with the findings in previous studies (4, 16, 17), the leading factors include eGFR and albumin urine concentration. Interestingly, we found that the inclusion of medication history, specifically drugs for the cardiovascular system and metabolism, and other laboratory tests such as hemoglobin levels improved the model’s performance in predicting the 5-year risk of CKD progression for diabetic patients.

Our developed CKD model outperformed the current clinical practice and the mainstream statistical models for predicting CKD progression. Clinicians use the eGFR to diagnose CKD in current practice. We developed a Cox regression model using only an eGFR to mimic the clinicians’ assessment. Additionally, we developed another Cox regression model using the features in Tangri’s paper (4), which was widely recognized and had a study design similar to ours. **Table 3** summarizes the performance of our models and other major models. Our model had the best performance in terms of area under the receiver operating characteristic (ROC) curve (AUC) value. The 95% confidence interval of our model’s AUC is 0.87 to 0.89, which is significantly higher than other statistical models.

**Table 3:** Performance of the different models.

Model	Method	Data	AUC
Our Model	XGBoost	Test data^1^	**0.88**
		6-month-shift test data^2^	**0.88**
Clinician	Cox regression	Test data	0.81
Statistical Model using features in Tangri et al. [2]	Cox regression	Test data	0.84
Tangri et al. [2]	Cox regression	Based on published paper	0.84
Lin et al. [3]	Cox regression	Based on published paper	0.86
Song et al. [4]	Gradient boosting	Based on published paper	0.83

^1^Test data are the cohort data before 1 January 2013, which contained 4,810 patients.

^2^Six-month shift-test data are the cohort data before 1 July 2013, which contained 4,590 patients. The 2 bold 0.88 are the AUC of our model, which are the highest compared to other models.

In addition, we tested the stability of our model’s performance on a new cohort by shifting 6 months. The new cohort consisted of the patients using the same exclusion criteria as in **Figure 2** but shifting the ending date to 30 June 2013 instead of 31 December 2012. The new cohort did not overlap with the study cohort discussed above. Our model predicted their risk of CKD progression in the next 5 years until 1 July 2018. As **Table 3** shows, our model’s performance was stable, indicating its potential to be generalized to new patients in different periods.

We have further validated our developed model clinically by comparison with clinicians tasked with predicting CKD progression clinically. We generated a new 2013 diabetes cohort of 1,000 patients as described in Methods and provided their features used by our model to 20 clinicians across all three polyclinics groups in Singapore (i.e., NHGP, NUP, and SHP). These 1,000 patients were not included in the original 2012 cohort that was used to develop the model or in the 6-month shift-test data. We asked the clinicians to assess the patients’ risk of CKD progression in the next 5 years and then compared the performance of their assessment and our model.

**Figure 4:**
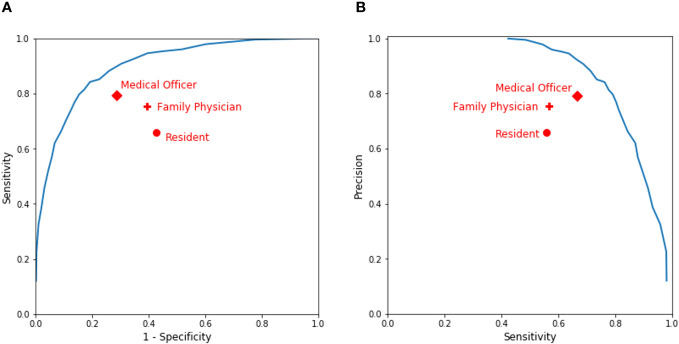
Performance of clinicians’ assessment vs. our model’s prediction. **(A)** A receiver operating characteristic (ROC) curve. **(B)** A precision–recall curve.

**Figure 4A** displays the receiver operating characteristic (ROC) of our model, in which the red dots represent the combinations of 1 – specificity and the sensitivity of the clinicians’ prediction. The area under the ROC curve (AUC) of the clinical validation data was still 0.88, which demonstrated, again, that our model could be generalized to new patients. **Figure 4B** shows the precision–recall curve of our model, in which the red dots represent the combinations of sensitivity and the precision of the clinicians’ predictions. The predictions by clinicians varied across seniority levels. Some clinicians did better than others in their assessments. However, our model outperforms all clinician subgroups because all the dots are under the curves in both **Figures 4A, B**. For example, our model was able to achieve a 0.74 sensitivity and a 0.81 precision in the clinical validation and it could also flexibly achieve the other balance along the curve in **Figure 4B**. Compared with clinicians of different seniority levels, our model predicted more accurately.

## 4 Discussion

CKD is a major complication and concern for diabetic patients, and two-thirds of new kidney failure cases are due to diabetes in Singapore. Identifying high-risk patients and intervening early will help prevent kidney failure, improve patient outcomes, and utilize hospital resources more cost-effectively. Our research contributes to identifying high-risk patients in the early stages of CKD. It is also different from previous studies in the following ways.

First, our project was the first national-level project on kidney diseases in diabetic patients. The study used data from the national cohort that covered the entire diabetic population in Singapore. The diabetic patients’ data, which were previously scattered among diabetic registries owned by different hospitals and the MOH, were collected and consolidated by the NDD. It contains the most complete datasets of diabetic patients in Singapore and is kept up to date with new data via the BRAIN platform. The national cohort represents the diverse population in Singapore, which includes different ethnicity groups from south and east Asia. As Ta et al. (11) introduced, the BRAIN platform runs on the existing infrastructure and draws the most current data in a timely manner from the source system. It has six core elements: (i) secure access channels, which enable the BRAIN platform to connect to multiple data sources; (ii) a national health identification service, which enables the BRAIN to uniquely identify people and their records received from different health domains; (iii) an enterprise terminology service, which enables the BRAIN platform to harmonize data across multiple source systems; (iv) a de-identification service, which supports the de-identification of patient data before presenting them to researchers or prediction tools, and the re-identification of patient data when needed; (v) user groups, which include data analysts, policymakers, and stakeholders; and (vi) tools for model and dashboard developments. Tools such as Python, R, Stata, and Tableau are available on the BRAIN platform. The BRAIN platform and NDD database are readily available to stimulate more research on diabetes in the future.

Second, our research identified new indicative factors that improve the prediction accuracy. In addition to the eGFR and the concentration of albumin urine, which have been proven to be predictive in previously developed models, our model found that medication history (e.g., drugs for the cardiovascular system and metabolism) and other laboratory tests (e.g., hemoglobin) may also play an important role in predicting CKD progression in diabetic patients. Some features that we used have correlations with each other, for example, urine albumin and the urine albumin-to-creatinine ratio. We kept both features because they can provide added value to predict CKD progression. Removing one will decrease the model’s performance on the validation set.

Third, our research developed a machine-learning algorithm that outperforms the current clinical practice and the mainstream statistical models in predicting CKD progression, as indicated in the results above. The technical validation of a 6-month shifted cohort also confirmed that our developed model is stable by showing the same good performance (with an AUC of 0.88) as the original base cohort. No calibration was needed when applying to different validation data, as our model was well trained using a representative national cohort in Singapore.

Fourth, our model performed better than clinicians in predicting CKD progression. More importantly, our model also reduced the variation of prediction among clinicians and provided a higher and more consistent accuracy.

**Figure 5:**
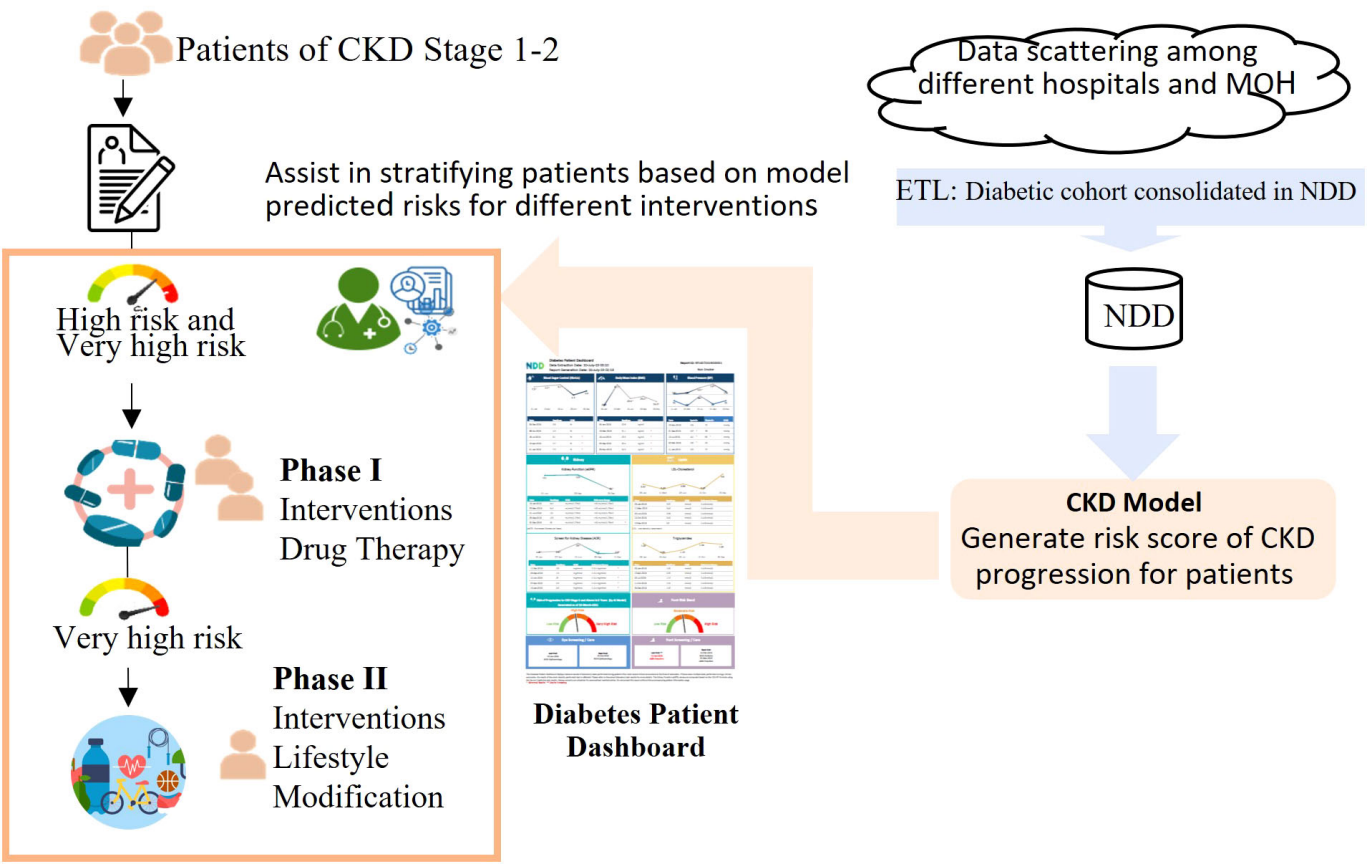
A workflow demonstrating how the HALT-CKD program is supported by the CKD predictive model. The cohorts of diabetic patients and their data were consolidated in the NDD (National Diabetes Database). The ETL jobs have been scheduled to automatically update the NDD data daily, transform the data into the model inputs, and trigger the CKD model to generate a risk score for the CKD stage 1 and 2 patients monthly. The risk score was banded into three levels and displayed on a diabetes patient dashboard. The users, such as clinicians, can view and print the dashboard results from the electronic health system and implement a more targeted early intervention for CKD stage 1 and 2 patients.

Fifth, our model was the first clinical model endorsed by the MOH and the HALT-CKD program in Singapore to be deployed nationwide by incorporating it into the HALT-CKD program, which was established to prevent and slow down the deterioration of CKD in Singapore, with many nephrologists from public hospitals in its committee. **Figure 5** shows how our model supports the HALT-CKD program to achieve more targeted early intervention. The cohorts of diabetic patients and their data were consolidated in the NDD. The ETL jobs have been scheduled to automatically update the NDD data daily, transform the data into the model inputs, and trigger the CKD model to generate risk scores for CKD1/2 patients once a month. The predicted score generated by the model can be flexibly banded into low, high, and very high risk to allow for the allocation of healthcare resources accordingly. The score is displayed on a diabetic patient’s dashboard together with other relevant data.

The two dashboards, as demonstrated in **Supplementary Figures 2**, **3**, were derived and integrated into the electronic health system. The Diabetes National Dashboard is a national-level dashboard that provides a snapshot of the diabetic profile. It provides cohort insights and supports decision-making for health policies and budget planning. The other dashboard is an individual-level dashboard, which incorporates the CKD model and consolidates key information for each patient, for example, laboratory tests and appointment information. It not only saves clinicians, who normally suffer from heavy clinical workloads, from carrying out the risk assessment of CKD progression but also provides improved accuracy when identifying the patients at high risk who may otherwise be missed given the time constraints of the consultation.

When patients come to visit clinicians, especially those in primary care who are the first line of care, the CKD stage 1/2 patients with a high risk of CKD progression will be flagged in the dashboard. The clinicians can view and print the dashboard results from the electronic health system, use it as a tool for patient coaching, and implement more targeted early intervention for them. For example, in the HALT-CKD program, there are two phases of intervention. In phase 1, patients with a high and very high risk will have more intensive drug therapy intervention, such as more frequent drug titration. In phase 2, which is more resource intensive, patients with a very high risk will be selected for lifestyle modification. Considering the 711,800 diabetic patients aged from 20 to 79 years old in Singapore in 2021^2^, approximately 474,500 diabetic patients, i.e., two-thirds of diabetic patients, will end up with kidney failure. By integrating the prediction model into the clinical workflow, these patients could benefit from early CKD intervention. The enhanced performance of the model enables the identification of more patients at a high risk of kidney deterioration. In general, the individual-level dashboard can fit into the current clinical workflow and enhance the clinicians’ ability to care for patients.

However, our study has a number of limitations, thus leaving room for future study. First, patients’ data from private health institutes are not available although they only constitute a small portion of the healthcare data of Singapore. This affects data completeness and might have influenced the model’s performance. Second, the impact of lifestyle factors, such as physical exercise and diet, has not been considered in the model. The model’s performance may be further enhanced by involving lifestyle factors. Third, our study is targeted at diabetic patients in Singapore, who mainly consist of Chinese, Malay, and Indian people. The model’s performance may decline, and the model should be retrained if applied to populations with different ethnicities.

In conclusion, our risk model of CKD progression for diabetic patients demonstrated its strength in prediction performance. It outperformed other predictive models and clinicians’ judgements in clinical validation. It has been endorsed by the MOH and the HALT-CKD to be deployed nationwide in Singapore to support clinicians in the assessment and medical treatment of patients in the early stages of CKD. Feedback will be collected from clinicians and the program committee to improve the predictive model further in the future. This project provided a successful example to demonstrate how an artificial intelligence (AI)-based model can be adopted to support clinical decision-making nationwide.

## Data availability statement

The original contributions presented in the study are included in the article/**Supplementary Material**. Further inquiries can be directed to the corresponding author.

## Ethics statement

The studies involving humans data were approved by the National Health Group Singapore. The studies were conducted in accordance with the local legislation and institutional requirements. Written informed consent for participation was not required from the participants or the participants’ legal guardians/next of kin in accordance with the national legislation and institutional requirements.

## Author contributions

ZF and WT contributed to the study design and main concept; ZF and ZW performed data analysis, developed the model, and drafted the paper. KC, MJ, and KP deployed the model. AY, GA, AL, CL, MF, and WC contributed to the data acquisition, designed the application of the model, and integrated the model into the national program. AL and CL revised the manuscript for important intellectual content. All authors read and approved the final draft before submission. WT is the guarantor of this work.

## Footnotes

1 The paper used 1-year medication data before 1 January 2013, and all past data of laboratory tests before 1 January 2013 from 1 January 2005.

2 Statistics is available at: https://www.diabetesatlas.org/data/en/country/179/sg.html
