# Supplementary material for: Development and deployment of a nationwide predictive model for chronic kidney disease progression in diabetic patients
Source: Front Nephrol. 2024 Jan 8;3:1237804. doi: 10.3389/fneph.2023.1237804 (PMC10800693; doi:10.3389/fneph.2023.1237804)
Supplement: Supplementary file 1 [file DataSheet_1.docx]

Supplementary Material

Development and Deployment of Nationwide Predictive Model for Chronic Kidney Disease Progression in Diabetic Patients

Zhiyan Fu, Zhiyu Wang, Karen Clemente, Mohit Jaisinghani, Ken Mei Ting Poon, Anthony Wee Teo Yeo, Gia Lee Ang, Adrian Liew, Chee Kong Lim, Marjorie Wai Yin Foo, Wai Leng Chow, Wee An Ta*

*** Correspondence:** Wee An Ta [andy.ta@ihis.com.sg](mailto:andy.ta@ihis.com.sg)

# Supplementary Text

The Kidney Disease Improving Global Outcome Guidelines (KDIGO) (1) is used to define the various chronic kidney disease (CKD) with slight variance on Stage 1 and 2. KDIGO requires 2 lab readings for CKD diagnosis, but many diabetes patients with estimated glomerular filtration rate (eGFR) $\geq$ 60 only had 1 lab reading. Considering the delay in CKD diagnosis, we therefore removed the criteria “2 tests > 90 days apart” from the definition on patients with CKD stage 1 and 2 (Table S1).

# Supplementary Figures and Tables

## Supplementary Figures


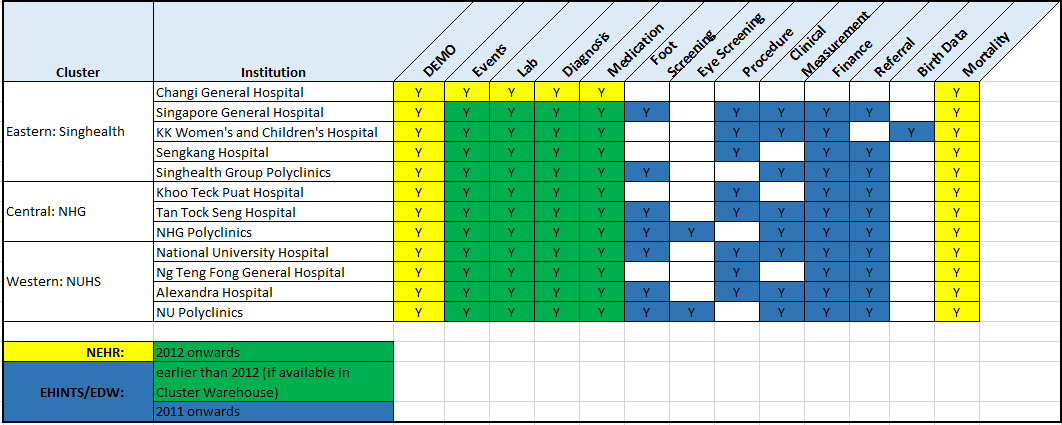


Supplementary Figure S1. Data Content in the National Diabetes Database

Supplementary Figure S2. Mock-up of Diabetes National Dashboard showing a general cohort profile, providing a national view on diabetic population and their related indicators


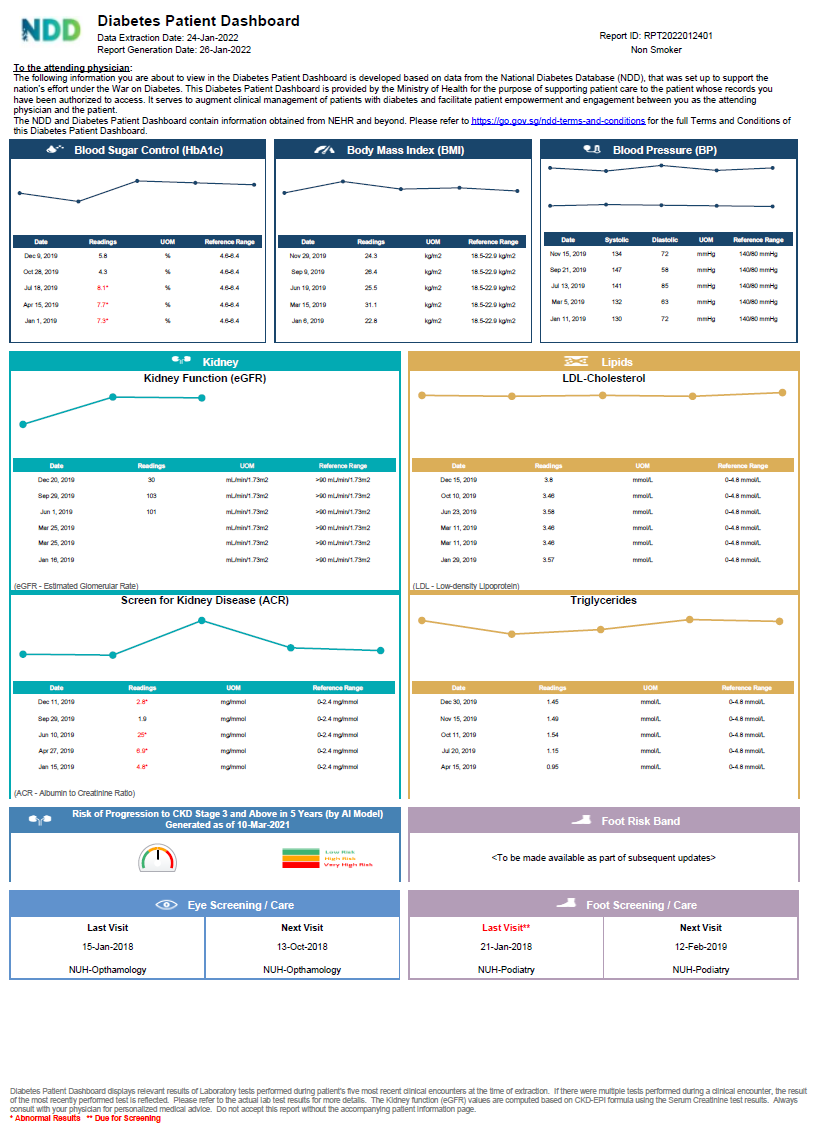


**Supplementary Figure S3. An example of Diabetes Patient Dashboard for a mock up patient**

## Supplementary Tables

| **CKD Stage** | **Definition** |
| --- | --- |
| **1** | eGFR ≥ 90 and ACR ≥ 3 *mg/mmol* |
| **2** | eGFR between 60 and 89 and ACR ≥ 3 *mg/mmol* |
| **3a** | eGFR between 45 and 59 in 2 tests > 90 days apart |
| **3b** | eGFR between 30 and 44 in 2 tests > 90 days apart |
| **4** | eGFR between 15 and 29 in 2 tests > 90 days apart |
| **5** | eGFR < 15 in 2 tests > 90 days apart |

Notes: eGFR is estimated glomerular filtration rate, ACR is albumin to creatinine ratio

Supplementary Table S1. Definition on Different Stages of Chronic Kidney Disease (CKD)

## Supplementary References

1. Levin A, Stevens PE. Summary of KDIGO 2012 CKD Guideline: behind the scenes, need for guidance, and a framework for moving forward. Kidney international. 2014;85(1):49-61.
